# Seroprevalence of circulating *Angiostrongylus vasorum* antigen and parasite-specific antibodies in dogs from Portugal

**DOI:** 10.1007/s00436-016-5001-x

**Published:** 2016-03-22

**Authors:** Ana Margarida Alho, Manuela Schnyder, Roland Schaper, José Meireles, Silvana Belo, Peter Deplazes, Luís Madeira de Carvalho

**Affiliations:** CIISA, Faculty of Veterinary Medicine, ULisboa, 1300-477 Lisboa, Portugal; Institute of Parasitology, Vetsuisse Faculty, University of Zurich, 8057 Zurich, Switzerland; Bayer Animal Health GmbH, 51368 Leverkusen, Germany; Unidade de Parasitologia Médica, Instituto de Higiene e Medicina Tropical, Universidade Nova de Lisboa, 1349-008 Lisboa, Portugal

**Keywords:** *Angiostrongylus vasorum*, Dog, ELISA, Seroprevalence, Epidemiology, Portugal

## Abstract

*Angiostrongylus vasorum* is a nematode that lives in the pulmonary arteries and right cardiac ventricle of domestic dogs and wild canids. It is increasingly being reported in several European countries and North America. This parasite induces inflammatory verminous pneumonia, causing severe respiratory disease in dogs. In some instances, coagulopathies, neurological signs and even death may occur. Scant data are available regarding the occurrence of *A. vasorum* in Portugal. Therefore, sera of 906 shelter dogs from North to South mainland Portugal were collected. ELISAs to detect *A. vasorum* circulating antigen and specific antibodies against this parasite were performed. A total of six dogs [0.66 %, 95 % confidence intervals (CI) 0.24–1.43] were positive for both *A. vasorum* antigen and antibody detection, indicating an active infection, and 12 dogs (1.32 %, CI 0.68–2.30) were *A. vasorum* antibody-positive only. Regions with antigen- and antibody-positive animals overlapped and were distributed over nearly all sampled areas in the country. This is the first large-scale ELISA-based serological survey for *A. vasorum* in dogs from Portugal. The endemic occurrence of *A. vasorum* in dogs from different geographical areas of Portugal is therefore confirmed.

## Introduction

*Angiostrongylus vasorum*, also known as the French heartworm, is described to have apparently spread in the last decade into previously uninfected areas (Helm et al. 2010). It is a potentially lethal parasite that resides in the heart and pulmonary arteries of dogs and wild carnivores, with gastropods acting as obligate intermediate hosts (Guilhon and Cens 1973). This nematode may cause a wide spectrum of manifestations in dogs, ranging from mild (or even absent) to severe forms that can be fatal. Respiratory signs (coughing and dyspnoea), bleeding disorders (haemorrhages) and neurological signs are the most frequent clinical features described. Howbeit, non-specific signs such as depression, weight loss, anorexia and exercise intolerance may also be present (Chapman et al. 2004; Wessmann et al. 2006; Koch and Willesen 2009). Such a wide variety of clinical signs makes it challenging to confirm or exclude a diagnosis of canine angiostrongylosis based exclusively on a clinical assessment.

A definite diagnosis can be reached using the Baermann method, through the detection of *A. vasorum* first stage larvae (L1), with the characteristic kinked tail, dorsal spine and notch feature (Guilhon and Cens 1973). FLOTAC, an improved flotation-based coproscopic method, also allows for the visualisation of *A. vasorum* L1 in faecal samples, with a good sensitivity (Schnyder et al. 2011a). However, due to prepatency, intermittent larval excretion and the possible occurrence of mixed lungworm infections, copromicroscopic techniques have limitations concerning sensitivity and specificity. Besides, by the time dogs start to be positive in Baermann or FLOTAC, damage to the lung parenchyma is already present, and recovery is more difficult (Guilhon and Cens 1969; Neff 1971; Dennler et al. 2011). Newly developed diagnostic techniques, such as PCR (Jefferies et al. 2009; Al-Sabi et al. 2010) and serological methods (Schnyder et al. 2011b; Schucan et al. 2012), have been developed to detect infected animals. Serological methods were shown to be highly suitable for both individual and massive screening of dog populations. In fact, *A. vasorum* serologies require single serum samples instead of repeated faecal samples and allows for rapid detection of infection, shortly before or contemporaneously with patency (Schnyder et al. 2015b).

Regarding the geographical distribution of *A. vasorum*, southern France (Guilhon and Cens 1969; Bourdeau 1993), south-east England and Wales (Jacobs and Prole 1975; Simpson and Neal 1982) and Denmark (Bolt et al. 1992) were traditionally considered areas with high endemic foci, while sporadic cases were diagnosed all over Europe. Nowadays, *A. vasorum* has a very heterogeneous distribution with reports suggesting the presence of endemic hotspots in many areas, namely in Croatia (Rajkovic-Janje et al. 2002), Italy (Della Santa et al. 2002; Guardone et al. 2013), Switzerland (Staebler et al. 2005), Germany (Staebler et al. 2005; Barutzki and Schaper 2009), Spain (Segovia et al. 2004; Mañas et al. 2005), Greece (Papazahariadou et al. 2007), Poland (Demiaszkiewicz et al. 2014), Slovakia (Miterpakova et al. 2014), Hungary (Schnyder et al. 2015a) and others. Several hypotheses have been raised to explain this possible expansion, such as increased movements of pet dogs and increased fox populations even in urban areas, suggesting that new areas are open to colonisation (Morgan et al. 2009).

In Portugal, knowledge concerning the current situation of *A. vasorum* infection in domestic and wild canids is poor. No studies conducted so far showed positive results, and no surveillance mechanisms are in place to assess its prevalence or geographical range. *A. vasorum* was first identified during the necropsy of one of 306 red foxes (*Vulpes vulpes silacea*) collected between 1970–1987, mostly from the coastal central and southern regions of Portugal, (Carvalho-Varela and Marcos 1993) and more recently, in the littoral centre of Portugal, with a prevalence of 16.1 % (Eira et al. 2006). Excluding foxes, *A. vasorum* was sporadically identified in domestic dogs, with three positive cases diagnosed in the last few years in the Lisbon area (Madeira de Carvalho et al. 2009, 2013; Nabais et al. 2014). A serological study using a commercial *A. vasorum* antigen kit (Angio Detect^TM^ Test, IDEXX Laboratories) tested negative on the 120 surveyed dogs from the Algarve region (Maia et al. 2015).

The present serological study aimed to increase the knowledge about the occurrence and geographical dispersion of *A. vasorum* infections in Portugal.

## Material and methods

A total of 906 shelter dogs randomly distributed from north to south of mainland Portugal were studied. All animals were stray dogs, and no information was available regarding previous preventive treatments. Blood samples (2–3 ml) were collected from the cephalic vein, and serum was separated by centrifugation and stored at −20 °C until use. Sera were tested at the Institute of Parasitology, Vetsuisse Faculty, University of Zurich, Switzerland, for the presence of circulating *A. vasorum* antigens using monoclonal and polyclonal antibodies in a sandwich ELISA, with a sensitivity of 95.7 % and a specificity of 94.0 %, as previously described (Schnyder et al. 2011b). Additionally, a sandwich ELISA (sensitivity 81.0 %, specificity 98.8 %) using *A. vasorum* adult somatic antigen purified by monoclonal antibodies (mAb Av 5/5) was used for specific antibody detection (Schucan et al. 2012). Test thresholds (Schnyder et al. 2013a) were regionally determined with 300 randomly selected samples based on the mean value of optical density (A_405_ nm) plus three standard deviations. All test runs included a background control, a conjugate control, three positive control sera from three experimentally infected dogs and two negative control sera from uninfected dogs.

The collected data were analysed using a geographical information system (GIS) program (RegioGraph 10, GfK GeoMarketing, Bruchsal, Germany) to visualise the regional distribution of collected and analysed serum samples and *A. vasorum* antigen- and/or antibody-positive samples. The locations of positive samples were displayed on maps with administrative and postcode boundaries based on the Portuguese four-digit postcode as points of reference. Excel 2007 for Windows (Microsoft Corporation, Redmond, USA) was used to calculate the prevalence values and their 95 % confidence interval (CI).

## Results

A total of 0.66 % of the dogs (*n* = 6, 95 % CI 0.24–1.43) were positive for both *A. vasorum* antigen and antibody detection, and 12 dogs (1.32 %, 95 % CI 0.68–2.30) were *A. vasorum* antibody-positive only. Additionally, a total of 1.99 % (*n* = 18, 95 % CI 1.18–3.12) of the dogs were *A. vasorum* antigen-positive only (Table 1).

**Table 1:** Serological results of 906 dog samples from Portugal tested for the presence of *A. vasorum* circulating antigens and for specific antibodies against *A. vasorum*

	Positive samples (*n*)	Percentage (%)	95 % confidence intervals
Antibody-positive	18	1.99	1.18–3.12
Antibody-positive only	12	1.32	0.68–2.30
Antigen-positive	24	2.65	1.70–3.92
Antigen-positive only	18	1.99	1.18–3.12
Antibody- and antigen-positive	6	0.66	0.24–1.43

**Fig. 1:**
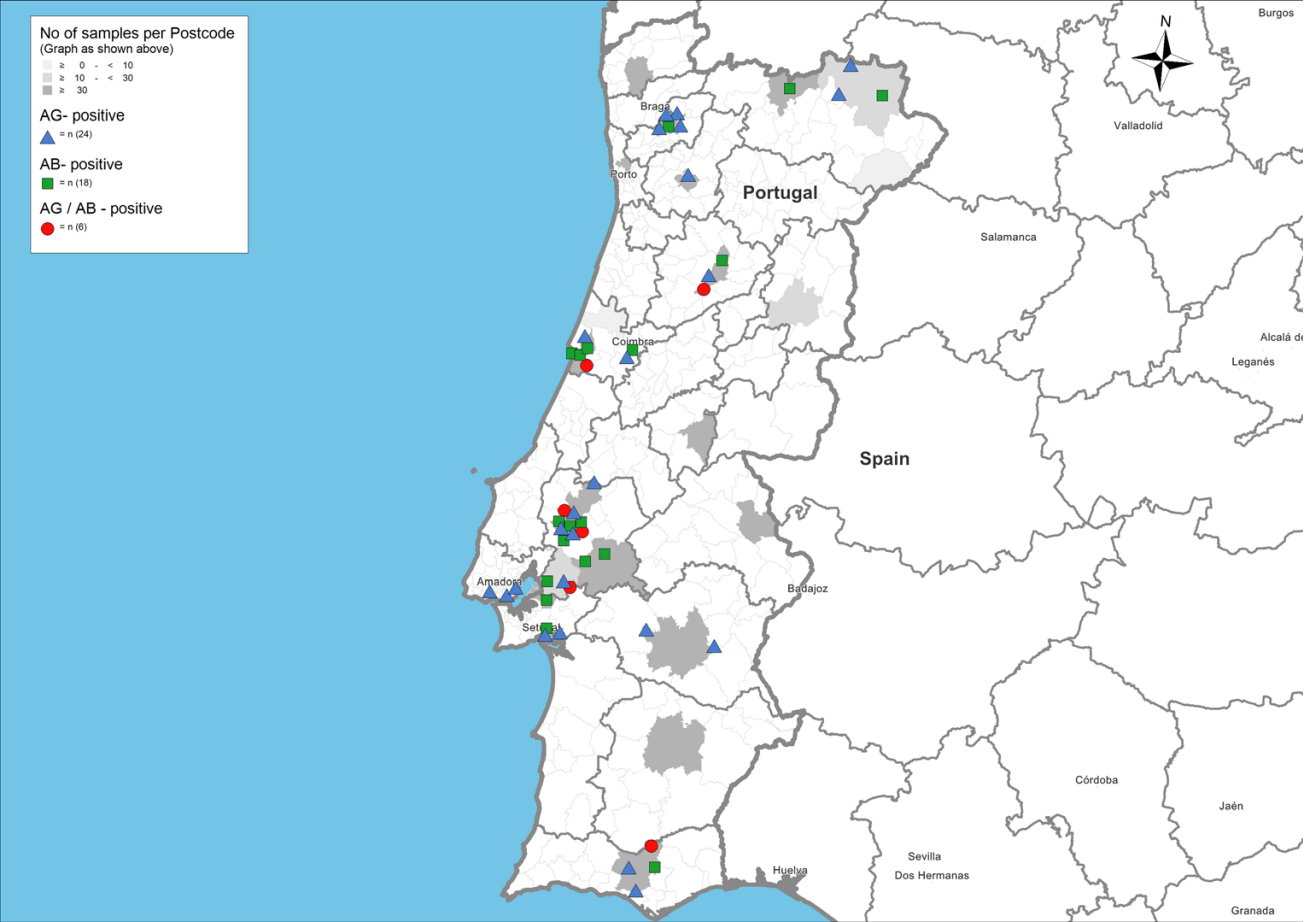
Occurrence of *Angiostrongylus vasorum* detected by ELISA in 906 dogs from Portugal. *Dark grey areas* represent the origin of the tested dog sera. Dogs positive for antigen and antibody are represented by *red dots*, dogs positive only for antibody are represented by *green squares* and dogs positive only for antigen are represented by *blue triangles*

Regions with antigen- and antibody-positive animals overlapped and were distributed over nearly all sampled areas in the country (Fig. 1).

## Discussion

Positive dogs for both ELISAs were detected in the north, centre and southern areas of Portugal, indicating an active infection widely distributed throughout the sampled area. The endemic occurrence of *A. vasorum* in dogs from different geographical areas of Portugal is therefore confirmed. Interestingly, *A. vasorum* was nearly absent in the central-eastern part of the country that borders Spain. Howbeit, the lack of evidence of *A. vasorum* in certain regions of Portugal does not ensure its non-existence, and thus, geographical location should not be used as the unique criterion to suspect or rule out this diagnosis.

With 0.66 % of the examined dogs being positive in both ELISAs, the prevalence in Portugal is apparently higher than that found for Germany (Schnyder et al. 2013a) or Poland (Schnyder et al. 2013b) and lower than in Hungary (Schnyder et al. 2015a), UK (Schnyder et al. 2013a) and Italy (Guardone et al. 2013), but not significantly. Nevertheless, it is important to highlight that this study surveyed shelter dogs, i.e., stray dogs usually not under any kind of prophylaxis and therefore frequently demonstrating higher parasitic prevalence (Alho et al. 2014).

Approximately 2 % of the dogs were positive for specific antibodies against *A. vasorum*, indicating parasite exposure: these dogs may have been sampled between 3 and 6 weeks after an *A. vasorum* infection, when antigen detection is still negative (first positive results between 7 and 11 weeks after infection), or the dogs were parasite-free but still antibody-positive after anthelmintic treatment or natural clearance of the infection (Table 1) (Schnyder et al. 2015b).

The results herein presented confirm the endemic occurrence of *A. vasorum* in dogs from different geographical areas of Portugal and may support the hypothesis of a gradual progression of *A. vasorum* in Portugal, since the prevalence of this parasite in red fox necropsies have also increased, from 0.3 % between 1970 and 1987 to 16.1 % between 2000 and 2006 (Carvalho-Varela and Marcos 1993; Eira et al. 2006). Several recent studies from other European countries such as Germany, Hungary, Switzerland, Great Britain or Italy illustrate the highly successful establishment of *A. vasorum* in the last decades. Although the reasons for this impressive success are not fully understood, a similar success within Portugal cannot be excluded. The occurrence of *A. vasorum* is linked to the presence of final hosts, among which red foxes represent powerful reservoirs and spreaders of this parasite, as indicated by higher prevalence in foxes compared to dogs (summarised in Koch and Willesen 2009). In fact, red foxes are the most widespread European wild canid (Otranto et al. 2015), greatly dispersed also all over the Iberian Peninsula (Macdonald and Reynolds 2008). This, in the absence of obvious geographic barriers, plays an important role in the expansion and establishment of *A. vasorum*, possibly explaining the high prevalence of this parasite detected in red foxes (*Vulpes vulpes*) in Portugal (Eira et al. 2006) and throughout Spain (Barbosa et al. 2005; Mañas et al. 2005; Gerrikagoitia et al. 2010). Also, the potential role of the wolf (*Canis lupus*) as a wild reservoir of *A. vasorum* can be mentioned based on a prevalence of 1.9–2.1 % reported in wolves from Spain (Segovia et al. 2001, 2007). Finally, the prevalence found in our study might be also explained by the results obtained in a pet owners’ questionnaire performed in Portugal, where it was shown that although the majority of the owners give antiparasitic drugs to their dogs, this often occurs at irregular and consequently ineffective intervals, with only 11.8 % of the dogs following the recommended endoparasitic treatment and only 28.4 % uninterruptedly protected throughout the year from canine vector borne diseases (European Scientific Counsel Companion Animal Parasites (ESCCAP 2016); Matos et al. 2015).

Interestingly, a simulation based on the observed distribution of *A. vasorum* in Europe and eco-climatic similarities predicted highly suitable areas in the north of Portugal and no suitability in the centre and southern part of the country (Morgan et al. 2009). Indeed, climate may play an important role in *A. vasorum* transmission, as the population dynamics and the activity of intermediate host species are highly dependent on temperature and moisture conditions. The north of Portugal is usually characterised by average low temperatures and high humidity in contrast to the south, where temperatures are frequently higher with lower humidity. Nevertheless, the slugs *Arion rufus* and *Deroceras laeve*, two gastropods known as intermediate hosts of *A. vasorum*, have already been described in distinct parts of the Portuguese territory (Grewal et al. 2003; Bank 2011).

To conclude, positive cases detected in such distinct areas of Portugal suggest that this parasite is now widespread in endemic foci over nearly the whole country. Since there have been only few studies in Portugal, it is not clear if the epidemiological situation in the country is stable or if it is moving.

Despite the complexity and challenges involved in diagnosing *A. vasorum* infection, when early detection and prompt-targeted therapy are undertaken, prognosis is good. Considering the impact of this disease on the health of affected dogs, it is thus important to increase knowledge concerning the epidemiological situation of this potentially fatal parasite. We believe that these results will be crucial to raise the awareness of veterinary practitioners, ensuring routine screenings of lungworms in dogs, and a well-timed diagnosis and treatment. Furthermore, we hope this data will contribute to highlight the importance of owner’s education in order to adopt behaviours that minimise the risk of infection.
